# Detection of Rehabilitation Training Effect of Upper Limb Movement Disorder Based on MPL-CNN

**DOI:** 10.3390/s24041105

**Published:** 2024-02-08

**Authors:** Lijuan Shi, Runmin Wang, Jian Zhao, Jing Zhang, Zhejun Kuang

**Affiliations:** 1 College of Electronic Information Engineering, Changchun University, Changchun 130012, China; shilj@ccu.edu.cn (L.S.); 210401139@mails.ccu.edu.cn (R.W.); 220401143@mails.ccu.edu.cn (J.Z.); 2 Jilin Provincial Key Laboratory of Human Health Status Identification Function & Enhancement, Changchun 130022, China; kuangzhejun@ccu.edu.cn; 3 Key Laboratory of Intelligent Rehabilitation and Barrier-Free for the Disabled, Changchun University, Ministry of Education, Changchun 130012, China; 4 College of Computer Science and Technology, Changchun University, Changchun 130022, China

**Keywords:** rehabilitation assessment, action recognition, Mediapipe, MPL-CNN, deep learning

## Abstract

Stroke represents a medical emergency and can lead to the development of movement disorders such as abnormal muscle tone, limited range of motion, or abnormalities in coordination and balance. In order to help stroke patients recover as soon as possible, rehabilitation training methods employ various movement modes such as ordinary movements and joint reactions to induce active reactions in the limbs and gradually restore normal functions. Rehabilitation effect evaluation can help physicians understand the rehabilitation needs of different patients, determine effective treatment methods and strategies, and improve treatment efficiency. In order to achieve real-time and accuracy of action detection, this article uses Mediapipe’s action detection algorithm and proposes a model based on MPL-CNN. Mediapipe can be used to identify key point features of the patient’s upper limbs and simultaneously identify key point features of the hand. In order to detect the effect of rehabilitation training for upper limb movement disorders, LSTM and CNN are combined to form a new LSTM-CNN model, which is used to identify the action features of upper limb rehabilitation training extracted by Medipipe. The MPL-CNN model can effectively identify the accuracy of rehabilitation movements during upper limb rehabilitation training for stroke patients. In order to ensure the scientific validity and unified standards of rehabilitation training movements, this article employs the postures in the Fugl-Meyer Upper Limb Rehabilitation Training Functional Assessment Form (FMA) and establishes an FMA upper limb rehabilitation data set for experimental verification. Experimental results show that in each stage of the Fugl-Meyer upper limb rehabilitation training evaluation effect detection, the MPL-CNN-based method’s recognition accuracy of upper limb rehabilitation training actions reached 95%. At the same time, the average accuracy rate of various upper limb rehabilitation training actions reaches 97.54%. This shows that the model is highly robust across different action categories and proves that the MPL-CNN model is an effective and feasible solution. This method based on MPL-CNN can provide a high-precision detection method for the evaluation of rehabilitation effects of upper limb movement disorders after stroke, helping clinicians in evaluating the patient’s rehabilitation progress and adjusting the rehabilitation plan based on the evaluation results. This will help improve the personalization and precision of rehabilitation treatment and promote patient recovery.

## 1. Introduction

Stroke is a common disease caused by haemorrhagic or ischaemic brain damage, and is one of the leading causes of death and physical disability [1]. The latest information provided by the World Stroke Organization (WSO) Global Stroke Fact Sheet 2022 [2] shows that, from 1990 to 2019, the incidence of stroke increased by 70.0%, the number of stroke deaths increased by 43.0%, and the disability-adjusted life-year prevalence of stroke was 143.0%. With the improvement in medical standards, the mortality rate of stroke has been continuously reduced. However, due to problems associated with an increasing and aging population, more and more people suffer physical disabilities after stroke. However, more than two-thirds of stroke patients experience upper limb movement disorders and dysfunction [3], which are characterized by muscle spasms, muscle weakness, and loss of movement coordination. In addition, post-stroke patients require six months of upper limb rehabilitation to restore basic motor functions [4]. Impairment of upper limb function is a common and serious dysfunction in stroke patients, with 32% of stroke patients having severe upper limb impairment and 37% having mild impairment. Most stroke patients can subsequently suffer from hemiplegia, which seriously affects the daily life of patients. Therefore, the rehabilitation of upper limb motor function is very important in the rehabilitation treatment of stroke patients. Patients with upper limb motor dysfunction can undergo rehabilitation training through neuroplasticity in the first three months, which is also essential to promote the recovery of upper limb motor function [5].

Due to the large number of patients and limited hospital resources, the rehabilitation training time for many patients is far less than six months, hindering full recovery of upper limb functions in affected patients when performing daily activities. This results in non-standard movements and difficulty in restoring motor memory, which will then lead to a decline in the patient’s self-care ability, seriously affecting the quality of life of stroke patients in addition to placing a significant financial burden on the patient’s family. To prevent the adverse consequences of upper limb motor dysfunction on the daily life of stroke patients, it is particularly important to provide timely and accurate scientific rehabilitation medical guidance for stroke patients. Therefore, conducting Fugl-Meyer upper limb assessment training and testing research on stroke patients bears very important social significance [6].

The algorithm for action recognition of stroke patients collects continuous video frames through common visual sensors, such as RGB cameras, and intelligently processes the video data to determine the current level of training of stroke patients [7]. Under the guidance of rehabilitation doctors, the collection of Fugl-Meyer upper limb rehabilitation exercise video data is completed. The intelligent processing of the collected data includes: classifying and labeling the collected original video data, and passing the labeled video data through Blaze-Pose [8] and Blaze-Hands to perform recognition of human bone joint points and hand joint points, and finally the deep learning algorithm completes Fugl-Meyer upper limb rehabilitation training action recognition. Since the conditions of stroke patients are different, the rehabilitation stages of the patients are also different. Evaluation of the rehabilitation effect of the patients needs to be carried out by experienced rehabilitation doctors. To this end, under the guidance of rehabilitation doctors, we selected scientific and effective assessment scales and rehabilitation movements, and used RGB cameras to collect Fugl-Meyer upper limb rehabilitation training movements.

Currently, commonly used algorithms for extracting key points of human skeleton include DensePose [9], Openpose [10], DeepPose [11], etc. When stroke patients undergo rehabilitation training, in order to meet the accuracy of the training process and better understand the patient’s rehabilitation situation, the real-time nature of the detection process needs to be considered. At the same time, during the Fugl-Meyer upper limb detection process, it is necessary to consider the movement of multiple parts of the body, such as shoulder joints, elbow joints, hand joints, and wrist joints. Therefore, Blaze-Pose and Blaze-Hands in Mediapipe [12] are selected to extract the feature information of the human body as a whole, including the human body and hands.

With the development of machine learning and the improvements in neural networks, this technology can apply many models for detecting human posture recognition and verify its effectiveness in action classification and behavior prediction. Convolutional neural network (CNN) [13] and long short-term memory neural network (LSTM) [14] have achieved breakthroughs in applications. CNN can extract features in data space, and LSTM can extract features in time. This article uses an LSTM-CNN behavior recognition and detection algorithm that can handle complex time series problems, and uses a two-layer network fusion algorithm to effectively perform upper limb rehabilitation and evaluate the effectiveness of training patient movements. First, OpenCV is used to segment the upper limb movement posture collected by the visual sensor into different frames, planarize the video data of standard actions, and connect the local features extracted by CNN and the long-distance features extracted by LSTM at the same time as a fully connected layer to ensure data flow. The continuity is used to improve the accuracy of the model, and the output results are used as movement standards for upper limb rehabilitation training for stroke patients. Finally, when patients undergo rehabilitation training, they only need to use visual sensors to evaluate the effects of current rehabilitation actions.

## 2. Related Works

Improvements targeting upper limb dysfunction aim to improve patients’ activities of daily living, functional independence and quality of life [15]. In addition to traditional therapies, the current use of robot-assisted technology, virtual reality technology and human posture recognition technology provides the possibility to help patients perform self- and remote rehabilitation training [16], which not only improves the scientific nature of patients’ home rehabilitation, but also promotes the assistance of rehabilitation therapists in patients’ upper limb rehabilitation training.

In robotic-assisted technology, Nam et al. [17] provided a musculoskeletal support for link extension in stroke patients by applying mechanical torque. Limb performance (measured by range of motion in joint extension) significantly improved when mechanical torque and NMES sensors were provided compared to limb performance without assistance, but the cable is driven by an electric motor with a gear/belt drive, resulting in increased complexity and weight of the entire assembly. This may result in greater physical burden on patients while wearing assistive robotic devices for autonomous rehabilitation exercises. Li et al. [18] proposed a new four-degree-of-freedom upper limb exoskeleton sensor compatible with upper limb motion for upper limb rehabilitation. In order to evaluate the compatibility of the exoskeleton, the authors conducted a large number of experiments, and the results showed that the introduction of passive joints can significantly reduce unnecessary forces on the exoskeleton in both static and dynamic modes. The bionic design of the upper limb exoskeleton is effective; therefore, it can be applied to upper limb rehabilitation training. The high cost of this assistive robot’s exoskeleton prevents it from being suitable for use in daily rehabilitation by most stroke patients. Silvia et al. [19] conducted a study to evaluate the usability of the MERLIN robotic system for the rehabilitation of upper limb stroke patients in a home setting and concluded that using the MERLIN system for upper limb home rehabilitation is safe and useful, actionable and motivating.

In traditional therapies, patients need to take the initiative to contact a rehabilitation doctor for guidance, but for low-income families, receiving guidance from a rehabilitation doctor in a hospital for rehabilitation training can impose a huge financial burden on the patient [20]. Moreover, the current shortage of rehabilitation doctors and the limitation of rehabilitation facilities in hospitals to meet the rehabilitation needs of a large number of patients have resulted in patients not being able to receive scientific guidance from rehabilitation doctors in a timely manner, making it impossible for movement disorders to be fully recovered. While the use of robot-assisted therapy can save healthcare resources, rehabilitation robots are overly intense and can pose safety issues for patients. Chen et al. [21] propose that robotic devices typically require a large physical space in living environments, and sometimes require appropriate conveniences such as tables and chairs to be installed. This is especially challenging for participants living in crowded spaces. Some robots can sometimes generate significant force, which can theoretically create safety issues when used unsupervised in the home.

Virtual reality technology can place patients in a virtual environment that integrates vision, hearing, and touch. They can perform rehabilitation training by wearing VR handles or helmets and other sensors to interact with the virtual scene, so that patients can achieve an immersive feeling. Lin et al. [22] conducted a randomized controlled trial to determine the effectiveness of virtual reality (VR) training on the emotional state, muscle strength, and motor function status of stroke patients at discharge. Paranes et al. [23] designed a prototype of a serious game using virtual reality to investigate how virtual reality can help stroke patients recover. Guo et al. [24] proposed an upper limb hand rehabilitation training system based on virtual reality, which can interact with virtual scenes and objects through collision detection and force feedback to complete the rehabilitation training process. Mekbib et al. [25] developed a VR system based on neuroscience principles that provides unilateral and bilateral upper limb (UE) training in a fully immersive virtual environment to help restore motor function after stroke. Given the high costs associated with VR equipment, it is relatively expensive for hospitals to purchase virtual equipment for the rehabilitation of stroke patients, thus increasing the medical costs for patients. Compared with using VR technology to obtain human body motion information by wearing related sensors, human posture recognition technology only needs to extract human skeleton joint point information through RGB cameras and other related visual sensors, and determine the patient’s rehabilitation actions by forming a human skeleton map. However, since upper limb movement disorders in stroke patients are also accompanied by hand movement disorders, hand rehabilitation testing is also very important. The Mediapipe used in this study can make up for this shortcoming. It can not only extract posture features using visual sensors, but also detect gesture features, so that the movement information of stroke patients can be more comprehensively detected when performing rehabilitation training.

In recent years, deep learning has been widely used in downstream tasks of great practical significance, such as computer-aided design tasks [26] and human posture recognition. Among them, human posture recognition is one of the most important basic tasks in computer vision and can be applied to tasks such as human action recognition, behavior analysis, and human–computer interaction. Human posture recognition technology has also been widely used in rehabilitation training for limb disorders. With the continuous improvement in human posture recognition technology, it has also been widely used in the field of intelligent rehabilitation. Through the human posture recognition algorithm, it can help patients carry out rehabilitation training intelligently, improve the intelligence of rehabilitation exercises, and at the same time provide a more complete comprehensive solution for the rehabilitation medical system. Rehabilitation training using human posture recognition technology is inseparable from visual sensors to collect human rehabilitation training movements. For video image data acquired by ordinary visual sensors, a new lightweight classification network is deployed on the visual sensor to complete the corresponding visual tasks. Yan et al. [27] combined Openpose and Kalman filter to track 2D gesture action sequences in RGB video streams, and proposed an efficient method for real-time continuous human rehabilitation action recognition to complete the rehabilitation training process in home scenarios. In order to help stroke patients carry out effective rehabilitation treatment, Tao et al. [28] used Openpose deep neural network to track key points of the human skeleton and complete activity trajectories and simulation modeling and verification of robot motion to meet the needs of rehabilitation doctors for the activity trajectories of rehabilitation robots. Li et al. [29] designed a home lower limb rehabilitation system based on a novel lightweight human posture estimation model, which can help patients perform rehabilitation training at home using their mobile phones. In work by Wu et al. [30], 3D motion of the human body in the lying position is obtained by combining 2D key points of the human body with depth information and coordinate transformation. A new method for human 3D pose estimation using RGB images and corresponding depth information proposed by Ji et al. [31] artificially improved the accuracy and real-time performance of rehabilitation action recognition. To better assist patients in long-term rehabilitation training in the home environment, a human rehabilitation action recognition algorithm Pose-AMGRU is proposed based on posture estimation and gated recurrent unit (GRU) network. The OpenPose pose estimation method is used to extract skeleton joint points from video frames. After preprocessing the pose data, key action features expressing limb movements are obtained. The attention mechanism is used to build a GRU network that integrates three-layer temporal features to achieve human rehabilitation action classification. The results show that it has high recognition accuracy. Shen et al. [32] proposed a lightweight real-time rehabilitation action recognition network (RARN) that uses skeletal sequences obtained through 2D pose estimation and constructed an intelligent assisted rehabilitation treatment system. A data set called Rehabilitation Movements for Degenerative Spondylosis (RDSD) was constructed for real-time movement evaluation of rehabilitation training. The results showed that the system achieved high-precision recognition. Cherry-Allen et al. [16] introduced human pose recognition while discussing the use of pose estimation to improve quantitative post-stroke rehabilitation assessment and demonstrated that automated video-based assessment can significantly improve post-stroke patient care and that using human gesture recognition technology can eliminate human factors (subjectivity of rehabilitation physician assessment) and infrastructure barriers, demonstrating easy-to-interpret outcome measures with direct clinical relevance using this technology, and increasing accessibility to groups without access to high-performance computing resources.

By comparing previous studies on using human posture recognition technology for rehabilitation training, we chose Mediapipe to extract key points of the human skeleton. Mediapipe [33] is an open source framework developed by Google. Mediapipe provides a range of pre-trained models and tools for computer vision and machine learning tasks such as pose estimation, face detection, gesture recognition, semantic segmentation, and more. Blaze-pose and Blaze-Hands provided in Mediapipe are lightweight models with high real-time performance and accuracy. Based on the above research, we used two lightweight models, BlazePose and BlazeHands, to simultaneously extract key points of the upper limbs and hands. This strategy aims to increase the richness of key point extraction in order to provide more features for the detection task of upper limb movement disorder rehabilitation training. By combining the BlazePose and BlazeHands models, we are able to capture the pose information of the upper limbs and hands simultaneously. The BlazePose model can accurately extract key points of the upper limbs, while the BlazeHands model focuses on extracting key points of the hands. Combining these two models can provide more comprehensive key point information, thereby providing more features for the recognition of rehabilitation training actions. This method of comprehensively utilizing multiple models helps to improve the representation ability of data and the robustness of the model, making the recognition of rehabilitation training actions more accurate and reliable. Through more comprehensive key point information, subtle changes in the upper limbs and hands in different movements can be captured, thereby providing more refined guidance and feedback for the evaluation and monitoring of rehabilitation training.

**Figure 1:**
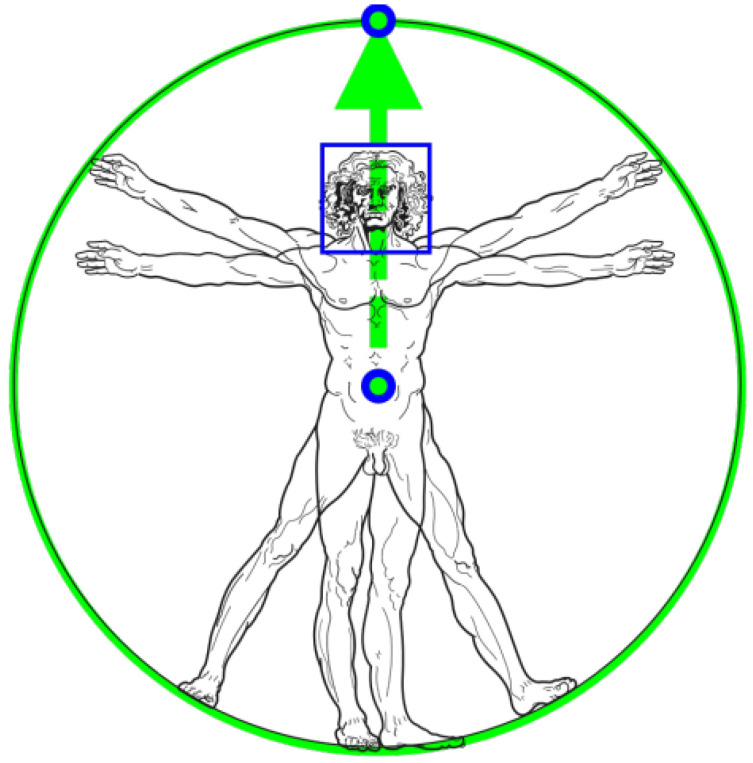
Vitruvian man bounding box.

Mediapipe uses inspiration from Vitruvian theory [34] to detect key points of the human body. By predicting the center point of a person’s hips, the radius of the body’s circumscribed circle, and the inclination angle of the straight line connecting the midpoints of the shoulders and hips, the tracking effect in complex situations is improved. Even in very complex situations, consistent tracking results can be obtained based on the proportion and orientation information of the hands and feet, as shown in Figure 1. This ensures the accuracy of patient assessment during rehabilitation training for upper limb movement disorders.

**Figure 2:**
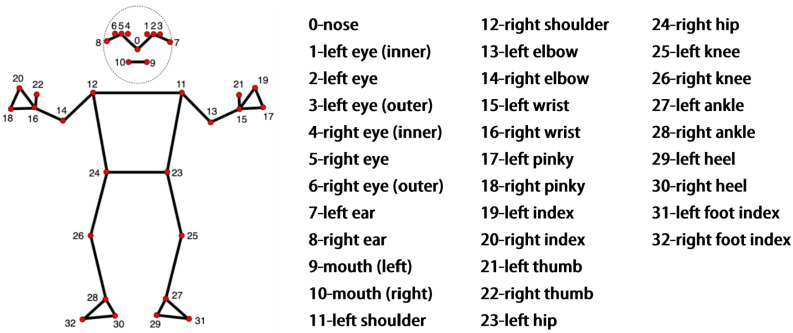
Vitruvian man bounding box.

**Figure 3:**
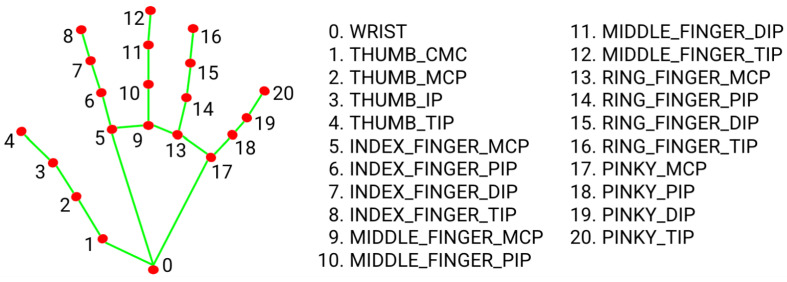
Vitruvian man bounding box.

Existing human body key point detection networks can accurately detect bone key points in various parts of the body, but often cannot be well combined with hand key points for overall detection. This results in stroke patients being unable to accurately assess the rehabilitation status of their hands when performing upper limb rehabilitation training, thus preventing doctors from obtaining comprehensive rehabilitation training information when performing upper limb rehabilitation assessment. To solve this problem, this study selected the Mediapipe keypoint extraction network. Compared with the 17 human body key points of the COCO topology, Mediapipe can simultaneously utilize machine learning to track 33 key points of human posture, as shown in Figure 2, and can detect 21 key points of the hand, as shown in Figure 3. When detecting human body key points and hand key points, Mediapipe uses a unified topology structure to extract posture and hand key point information.

## 3. Methodology

### 3.1. Mediapipe Joint Point Extraction

**Figure 4:**
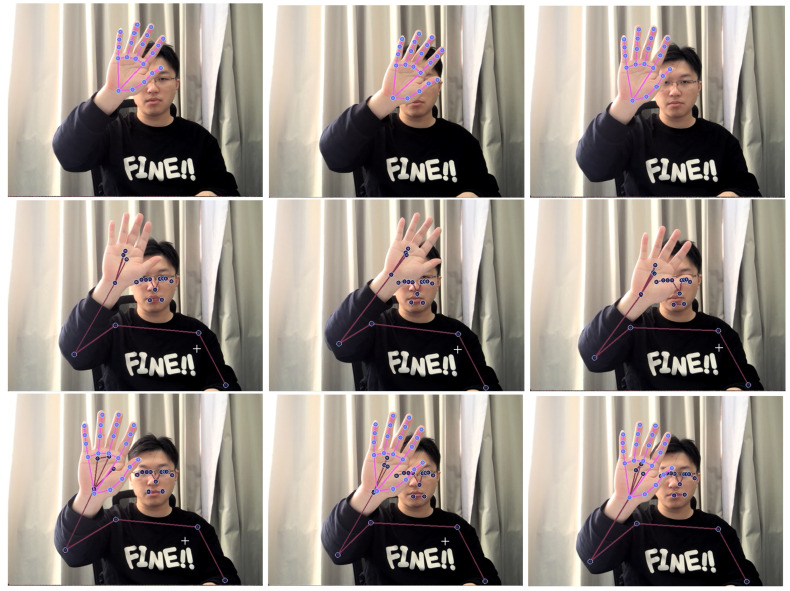
Detection of Blaze-Pose, Blaze-Hands and overall detection. The three pictures in the first row represent the hand joint points detected by Blaze-Hands; the three pictures in the second row represent the upper limb joint points detected by Blaze-Pose; the third picture represents the joint points of the upper limb and hand overall detection.

Using the Blaze-Pose and Blaze-Hands models in MediaPipe, efficient and accurate human joint point detection and hand joint point detection can be achieved. The detection display is shown in Figure 4.

The Blaze-Pose model is based on deep learning technology, and with the help of advanced neural network architecture and training strategies, it can quickly and accurately estimate the three-dimensional joint point position and posture information of the human body in real-time scenes. The Blaze-Hands model focuses on the detection of hand joint points and achieves highly accurate inference of hand postures and gestures by combining deep learning and computer vision algorithms. MediaPipe is used to perform pose estimation and gesture recognition in parallel to achieve multi-task key point detection tasks. Among them, pose estimation aims to extract the three-dimensional joint point positions and posture information of the human body from the input video, while gesture recognition focuses on accurately inferring the joint point positions and gesture movements of the hand. Therefore, in the process of testing the effect of rehabilitation training for upper limb motor dysfunction, the joint points of the human body and the joint points of the hand are extracted at the same time. It can provide rehabilitation doctors with more complete rehabilitation training information, making it more accurate for rehabilitation doctors to evaluate rehabilitation effects.

### 3.2. LSTM-CNN Rehabilitation Action Recognition Network

CNN is a deep learning model specially used for image processing. CNN can automatically learn and extract features in images and be used for tasks such as classification, detection, and segmentation. In image classification, CNN usually consists of a series of convolutional layers, pooling layers and fully connected layers. Convolutional layers are used to extract local features of the image. The pooling layer is used to reduce the dimensionality of the feature map, and the dropout layer can prevent over-fitting problems. The fully connected layer is used to map the extracted features to category labels. Agnieszka Szczęsna et al. [35] applied CNN to analyze functional upper limb movement patterns, To find the differences present in the upper limb movement patterns, the movement characteristics of the dominant and non-dominant upper limbs of healthy participants were compared with the movement characteristics of the flaccid and non-flaccid upper limbs of stroke participants. A new CNN application is proposed for recognition of motion data with two different class label configurations.

**Figure 5:**
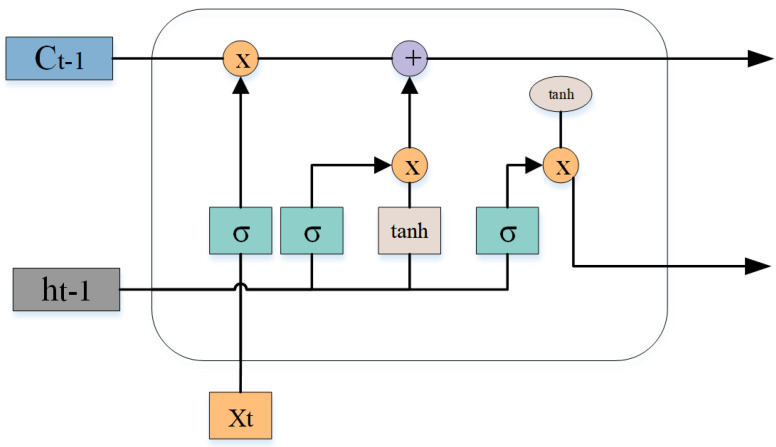
LSTM workflow diagram.

For action recognition tasks, in addition to spatial features, it is also necessary to model the temporal information of actions. However, traditional CNN structures are not specifically designed for temporal modeling and cannot fully capture the temporal dependencies in action sequences. Recurrent neural networks (RNN) have great advantages in processing time series information. However, due to the lack of spatial construction of RNN, and the network structure of RNN, the problems of gradient explosion and gradient disappearance are unavoidable, making RNN ineffective for processing time series information. To solve this problem, this study uses long short-term memory neural network (LSTM) to process temporal skeleton information. LSTM is a special recurrent neural network. The use of LSTM can ensure the effective long-term storage of information [36] and solve the long-term dependency problem existing in recurrent neural networks. The memory unit is introduced into the LSTM network, which can remember important information in long-term time series through the storage unit of the self-renewal mechanism, as shown in Figure 5. When LSTM processes time series data, it needs to pass through three joint gates: input gate, output gate and forget gate. It also includes long and short memories, and calculates the mapping from the input sequence vector to the output probability vector by using the following formula:

input gate:

(1) $$i_{t}=\sigma \left(W_{f}\cdot \left[h_{t}-1,x_{t}\right]+b_{f}\right)$$

output gate:

(2) $$o_{t}=\sigma \left(W_{o}\cdot \left[h_{t}-1,x_{t}\right]+b_{o}\right)$$

forget gate:

(3) $$f_{t}=\sigma \left(W_{f}\cdot \left[h_{t}-1,x_{t}\right]+b_{f}\right)$$

(4) $$\tilde{C_{t}}=\tan\left(W_{c}\cdot \left[h_{t}-1,x_{t}\right]+b_{c}\right)$$

Two memories:

Long memory:

(5) $$C_{t}=f_{t}*C_{t-1}+i_{t}*\tilde{C_{t}}$$

Short memory:

(6) $$h_{t}=o_{t}*\tanh\left(C_{t}\right)$$

From time t=1 to N to iterate, LSTM will map the input sequence vector $X=\left(X_{1},X_{2},X_{3},\cdots ,X_{n}\right)$ to the output through the formula. Among them, $C_{t}$ is the current memory unit, σ is the sigmoid activation function, tanh is the tanh activation function, *W* represents the weight matrix, $\tilde{C_{t}}$ is the current cell status at this moment. $f_{t}$ is the information that determines the discarding.

Long short-term memory network (LSTM) and convolutional neural network (CNN) each have their own characteristics. By combining LSTM with CNN, LSTM’s sequence modeling capabilities and CNN characteristics extraction capabilities, this helps reduce the excessive risk of model fitting It can also be further improved by adding regular techniques such as Dropout.

When the time sequence data are input into the LSTM-CNN network, the model can effectively capture the time correlation and space correlation in timing data, and gradually extract higher-level features. This helps to understand the abstract level of data and improve the expression of the model. The front end of the model proposed in this article is the LSTM part, and the back end is the CNN part, which combines the features extracted to output the final result.

## 4. Experiments

### 4.1. Fugl-Meyer Upper Limb Rehabilitation Training Standards

**Figure 6:**
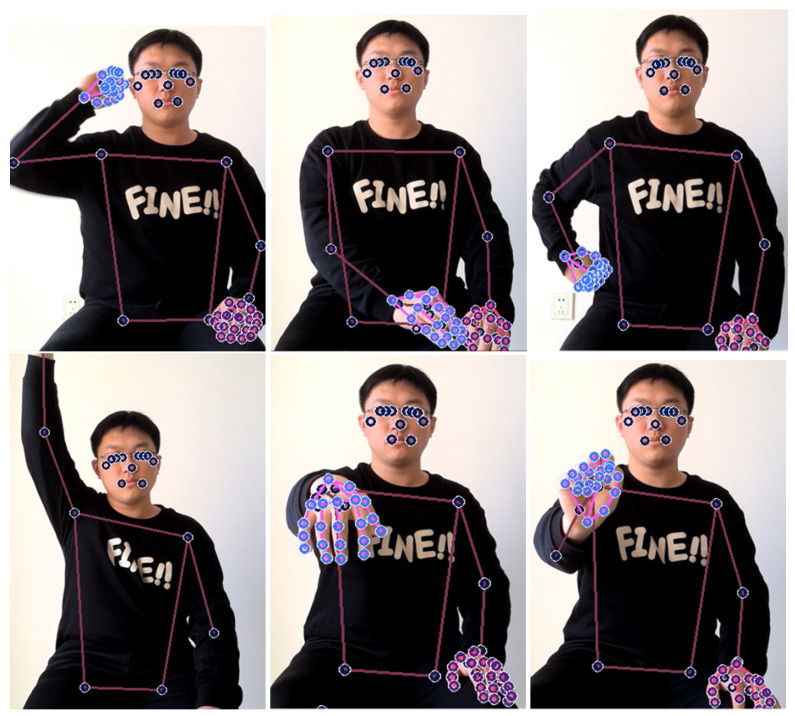
FMA standard posture detection. The three pictures in the first row represent the coordinated movement of flexor muscles, the joint movement of extensor muscles and the accompanying joint movement, respectively; the three pictures in the second row represent isolated movement, wrist stability and affected movement, respectively.

When selecting movement characteristics for upper limb rehabilitation training, the selection needs to be based on the Fugl-Meyer Upper Limb Functional Assessment Scale (FMA) under the guidance and supervision of a clinician. FMA test items generally include movements of the shoulder joint, elbow joint and wrist joint. At the same time, we added hand movements based on the FMA test items to meet the needs of action feature selection in this experiment. Some FMA actions are shown in the Figure 6 below.

Since the collected movements are continuous, such as the coordinated movement of flexor muscles, the movement process is to touch the ear on the same side with your hand, and then move your hand to touch the knee joint on the opposite side. The details of FMA rehabilitation training movements are shown in the table below.

### 4.2. Dataset Introduction

Since there are currently no publicly available action recognition and Fugl-Meyer video data sets for upper limb sports rehabilitation training, we completed the Fugl-Meyer upper limb rehabilitation training through these 10 subjects. A total of ten neurologically normal adults, including five men and five women, participated in the study and underwent Fugl-Meyer upper limb motor rehabilitation training for data collection. They had no orthopedic or neurological conditions that limited upper limb movement, no cognitive or speech impairments, and an average age of 48.4 years. The age range is generally consistent with the age of patients undergoing rehabilitation for upper limb movement disorders after stroke and those undergoing rehabilitation for upper limb movement disorders. All participants completed corresponding rehabilitation actions under the guidance of doctors with experience in post-stroke movement disorder assessment and clinical treatment. Videos of all movements of each subject completing five rehabilitation training postures were recorded. This experiment mainly collects motion signals from standard rehabilitation training. These signals are under the guidance and supervision of clinical rehabilitation physicians to ensure the accuracy and scientificity of rehabilitation actions. The original video is in MP4 format, and all poses are completed within a distance of 2–3 m in front of the camera. In an indoor environment, all poses were collected within 10 s at 30 frames per second (FPS), with each subject repeating different rehabilitation training actions 2 times and 5 times each, for a total of over 42,000 frames. A description of the collected dataset is shown in the figure, including the number of people and videos for each pose. The data set is divided into a training set and test set according to a 4:1 ratio. The introduction of the data set is shown in Table 1 and Table 2 below.

### 4.3. Model Design

**Figure 7:**
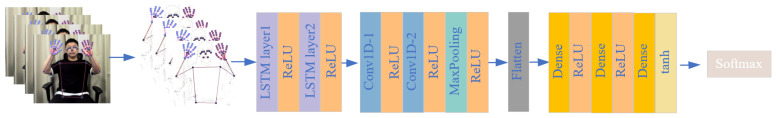
LSTM-CNN model architecture.

This experiment entails building an action recognition model based on the LSTM model. The LSTM model can better learn action feature information by adding CNN, thereby improving the performance and training effect of the model. The LSTM-CNN model as a whole consists of eight layers of sequences. The first two layers of the model are composed of LSTM. The first layer of LSTM has 64 hidden units and the input shape is (30, 258), which means that the input data has 30 time steps and 258 features, and the complete output sequence is returned. The second layer of LSTM has 128 hidden units, and the activation functions used in both layers are Relu. It is then used to extract spatial features through CNN. The CNN layer contains two one-dimensional convolution layers and a maximum pooling layer. The first one-dimensional convolution layer contains 64 3 × 3 convolution kernels. The second layer of one-dimensional convolution contains 128 3 × 3 convolution kernels. The maximum pooling layer uses a pooling window of size 2 to perform a maximum pooling operation on the input sequence. The activation function used is Relu. After the flattening layer, the input data are converted from multi-dimensional tensors to one-dimensional vectors. Then through the Dropout layer, a part of the input data is randomly discarded with a dropout rate of 0.2 to reduce overfitting. Afterwards, through three fully connected layers with 64, 32 and 4 hidden units, the activation function of the first two layers is ReLU and the third layer is the hyperbolic tangent function (tanh). Finally, the classification results are output through softmax. Its network model structure is shown in Figure 7.

### 4.4. Experiment Analysis

**Figure 8:**
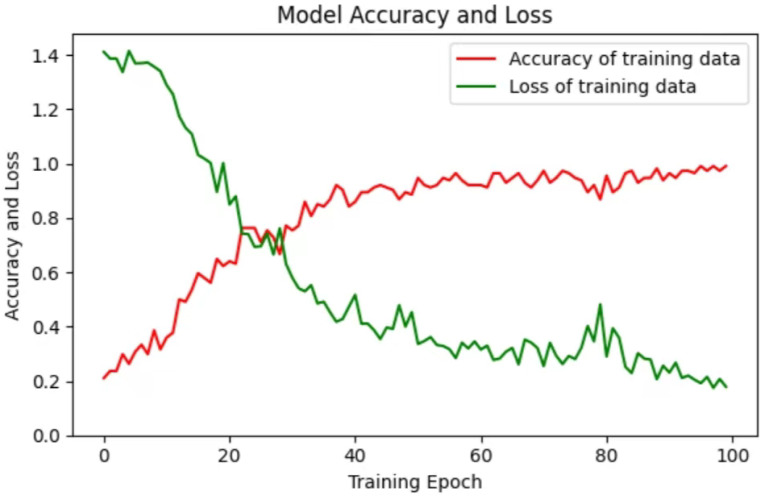
Model loss and accuracy iteration process.

In order to ensure that the detected upper limb movement disorder rehabilitation training movements are scientific and effective, this study used the Fugl-Meyer Upper Limb Rehabilitation Assessment (FMA) standard movements. These movements include flexor co-movements, extensor co-movements, accompanying co-movements, isolation movements, wrist stability and hand movements. The data set was collected using BlazePose and BlazeHands according to the standard movements in FMA. Compared with the simple extraction of upper limb key points, this study also extracted hand key points. The collected data set is input into the LSTM-CNN model to detect the standard actions of FMA. The LSTM-CNN model consists of two LSTM layers. Through the dimensionality reduction operation of the first LSTM layer, the model can extract important feature representations from the input sequence and reduce the computational complexity of subsequent LSTM layers. After dimensionality upscaling by the second layer, the high-dimensional input is mapped to a low-dimensional hidden state representation, which helps capture key patterns and long-term dependencies in the sequence. And it reduces the amount of calculation at each time step, thereby improving the efficiency of training and inference to a certain extent. CNN consists of two convolutional layers and a max pooling layer. The convolutional layer extracts local features at different locations through a 3 × 3 convolution kernel, and learns different filter weights to capture different patterns and features in the input sequence. Max pooling helps the model extract the most salient and important features from the input data. It is converted into a one-dimensional vector in the Flatten layer, and the ReLU method is used to improve the generalization ability between networks. Afterwards, the previous features can be further combined and transformed through the fully connected layer model to obtain higher-level features and generate feature representations. In the last fully connected layer, the learned features are mapped to the four-dimensional output space, and finally the Softmax output is used to complete the classification task. In the model, the learning rate of each parameter is adaptively adjusted through the Adam algorithm. The learning rate can be automatically adjusted according to the gradient change of the parameters, so that the learning rate can adapt to the update needs of different parameters. By using the Adam algorithm, the training process can converge faster, making it easier to achieve good performance than manually setting the learning rate. And use categorical_crossentropy to measure the difference between the model prediction results and the real labels, prompting the model to better learn the differences between categories. By minimizing this loss function, the model can better distinguish feature differences between different categories and improve the accuracy of classification tasks. The loss and accuracy of classification results training and testing change with the iteration curve as shown in the Figure 8.

During the upper limb rehabilitation training process, the visual sensor receives real-time video information and generates a human skeleton information sequence through pre-processing steps such as Blaze-Pose and Blaze-Hand. This skeletal information sequence is real-time and continuous, and it contains key joint position and motion information of human posture and hand movements. In order to better process this sequence of bone information, we used the LSTM-CNN model for detection. LSTM (Long Short-Term Memory) is a variant of Recurrent Neural Network (RNN) suitable for sequence data, capable of capturing temporal dependencies in the sequence. CNN (Convolutional Neural Network) can extract spatial features. By combining LSTM and CNN, our model is able to effectively process skeletal information sequences and detect and classify actions. The LSTM model can model the temporal information in the sequence and capture the evolution of the action. The CNN model can extract spatial features in the skeletal information sequence, such as joint positions and motion patterns. In addition, in order to further improve the performance of the model, we propose the MPL-CNN model. This model pays special attention to feature information in time and space when processing skeletal information sequences. This means that we focus not only on the temporal evolution in the sequence, but also on the spatial relationships and motion patterns between different joints.

By using the MPL-CNN model, we are able to more comprehensively analyze and understand the skeletal information sequence in upper limb rehabilitation training. This method of comprehensively considering temporal and spatial features helps improve the accuracy and robustness of the model for action recognition and analysis, thereby better supporting the upper limb rehabilitation training process. This study uses a self-made upper limb posture data set, including shoulder joints, elbow joints, wrist joints and gestures, and inputs the data into the model for training. The evaluation index calculation formula is as follows:

(7) $$Accuracy=\frac{TP+TN}{TP+FN+FP+TN}$$

(8) $$Precision=\frac{TP}{TP+FP}$$

(9) $$Recall=\frac{TP}{TP+FN}$$

(10) $$F1=\frac{2\cdot Precision\cdot Recall}{Precision+Recall}$$

**Figure 9:**
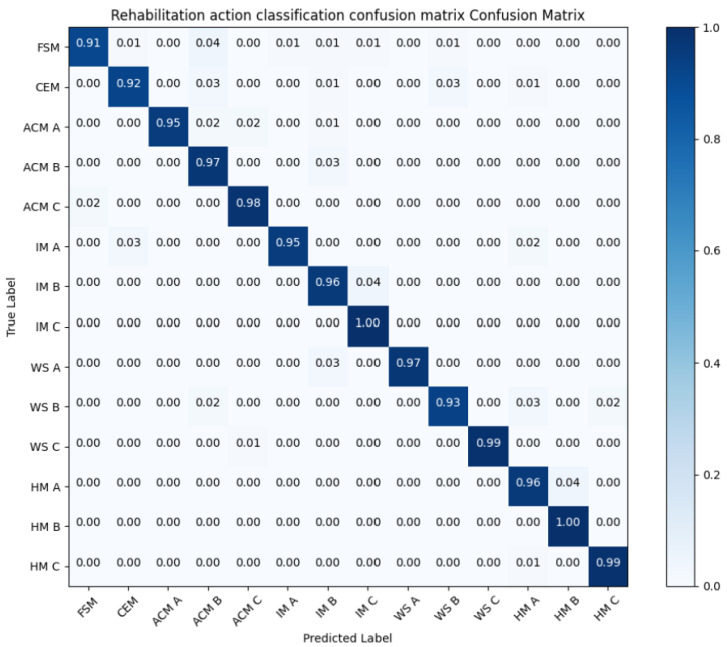
Confusion matrix of standard movements of Fugl-Meyer upper limb movement disorder rehabilitation training.

The model’s detection of rehabilitation training actions is shown in the Table 3 and Figure 9. Upper limb rehabilitation training movements recognized using MPL-CNN including co-movement of flexor muscles, co-movement of extensor muscles, accompanied by co-movement, wrist stability and hand movement. The average classification accuracy is 97.54%, and the recall rate and F1-score are both above 90%. proving that the MPL-CNN model has high efficiency and reliability in upper limb movement disorder rehabilitation training detection tasks. It can meet the requirements for detecting the effects of rehabilitation training on stroke patients. The accuracy of MPL-CNN in classifying upper limb rehabilitation movements is 99.22%. The upper limb movement detection network structure based on MPL-CNN proposed in this study has an accuracy increase of 2% compared with the method proposed by Ashwini [38]. Compared with machine learning methods [39], the accuracy has also been improved to a certain extent. Compared with spatiotemporal CNN [40], the recognition accuracy is increased by 3.44%. To sum up, the upper limb movement disorder detection model proposed in this article based on MPL-CNN can use Mediapipe to extract action information only by using an RGB camera. It can achieve good real-time performance while also achieving better detection accuracy.

### 4.5. Ablation Experiment

In order to explore the impact of adding three modules to MPL-CNN (1. Introduction of LSTM, 2. Introduction of CNN convolution module, 3. Adding Dropout to optimize LSTM-CNN) on the final performance, this article uses the upper limbs to conduct ablation experiments on the skeletal key point frame data obtained by Mediapipe, and combines different modules to calculate the accuracy. In order to better quantify the impact of each module on model performance, in addition to category accuracy, the recognition accuracy of different upper limb movement disorder rehabilitation training actions was also increased. In order to balance the differences in actions, different action types may have different difficulties and characteristics, resulting in differences in recognition accuracy. By taking the average P of the recognition accuracy of multiple action types, we balance the impact of each action type and reduce the impact of certain action types on the overall performance evaluation. The test results are shown in Figure 4. It can be seen from the analysis in Table 4 that through the addition of various network modules, the accuracy rate has been significantly improved, reaching 99.22%. Compared with single network performance, the maximum improvement is 15.59%. Compared with a single network, P’s performance can be improved by up to 6.25%. The results show that adding three modules to the MPL-CNN model improves the performance of upper limb movement disorder rehabilitation training action detection, proving its effectiveness.

## 5. Conclusions

This study is based on the use of Mediapipe and LSTM-CNN for motion detection in rehabilitation training of stroke patients. In this process, we employed the Fugl-Meyer standard movements for upper limb motor function assessment and established a standard movement data set. Mediapipe is used to extract feature information of standard actions, including upper limb posture feature information extracted by Blaze-pose and gesture feature information extracted by Blaze-Hands. Both are under the Mediapipe framework, so the upper limbs can detect postures and gestures at the same time, ensuring the richness of key point information during standard action detection for upper limb movement disorder rehabilitation training. The MPL-CNN model was proposed to identify standard rehabilitation training movements for upper limb movement disorders, and a detection experiment for upper limb rehabilitation training movements was conducted. Experimental results show that the recognition accuracy of the MPL-CNN model is improved in identifying standard rehabilitation training movements for upper limb movement disorders. The standard movements for the Fugl-Meyer upper limb motor function assessment all reach over 95%. By observing the average accuracy of each rehabilitation movement category, reaching 97.54% ensures the stability of the model in different action categories, proving that the model can be effectively used for the detection of upper limb rehabilitation training in stroke patients.

Based on the characteristics of Mediapipe’s real-time and cross-platform support, it can be directly deployed on mobile devices, and our later work will mainly focus on deploying the MPL-CNN model on mobile devices for experimental testing to determine the practicality of our model. After being deployed on mobile devices, patients can observe their rehabilitation training effect at any time through mobile devices, save their rehabilitation training records, and provide rehabilitation information to rehabilitation doctors, so as to provide more scientific and effective rehabilitation training guidance to patients.

## Figures and Tables

**Table 1:** Fugl-Meyer upper limb rehabilitation exercise score [37].

Types of Rehabilitation Exercises	Movement	Standard Action	Label
Flexor synergistic movement	Touch the affected upper limb to the ipsilateral ear.	The shoulder joint is raised and retracted, abducted to 90° and externally rotated, the elbow joint is fully flexed, the forearm is fully supinated.	FSM
Common extensor muscle movement	Touch the opposite knee joint with the affected hand	Adduct the shoulder joint, internally rotate, extend the elbow joint, pronate the forearm.	CEM
Accompanied by common movement	-	1. The patient touches the waist with the back of the hand.	ACM A
		2. Elbow straight, shoulder joint flexed 90°, shoulder 0°.	ACM B
		3. Bend the elbow 90° to complete the pronation and supination movement of the forearm.	ACM C
Isolation movement	-	1. Abduct the shoulder joint to 90°, straighten the elbow and do forearm pronation movement.	IM A
		2. Bend the shoulder joint forward and raise the arm overhead, elbow straight.	IM B
		3. Flex the shoulder between 30° and 90° to complete the pronation and supination movement of the forearm.	IM C
Wrist stability	-	1. Shoulder 0°, elbow flexion 90 degrees, complete wrist dorsal flexion 15 degrees and alternate wrist flexion and extension movements	WS A
		2. Shoulder flexed 30°, elbow extended, forearm pronated to complete 15 degrees wrist dorsiflexion and alternate wrist flexion and extension movements	WS B
		3. Circular motion of the wrist joint	WS C
Hand movement	-	1. Finger joints are at 0°, and the fingers are flexed and extended together, forming a hook shape.	HM A
		2. Finger joint 0°, thumb adducted.	HM B
		3. Flex your fingers to make a grasping motion.	HM C

**Table 2:** Dataset introduction.

NO.	Posture	Number of People	Number of Videos
1	Flexor synergistic movement (FSM)	10	100
2	Common extensor muscle movement (CEM)	10	100
3	Accompanied by common movement A (ACM A)	10	100
4	Accompanied by common movement B (ACM B)	10	100
5	Accompanied by common movement C (ACM C)	10	100
6	Isolation movement A (IM A)	10	100
7	Isolation movement B (IM B)	10	100
8	Isolation movement C (IM C)	10	100
9	Wrist Stability A (WS A)	10	100
10	Wrist Stability B (WS B)	10	100
11	Wrist Stability C (WS C)	10	100
12	Finger movement A (HM A)	10	100
13	Finger movement B (HM B)	10	100
14	Finger movement C (HM C)	10	100
	Total number of videos		1400

**Table 3:** MPL-CNN recognizes rehabilitation training action results.

Rehabilitation Type	Precision	Recall	F1-Score
FSM	97.92%	91.67%	94.69%
CEM	95.43%	92.71%	94.05%
ACM A	100%	94.79%	97.32%
ACM B	91.46%	96.88%	94.09%
ACM C	97.66%	98.96%	98.30 %
IM A	99.77%	95.83%	97.76%
IM B	94.83%	96.88%	95.84%
IM C	95.67%	100%	97.78%
WS A	100%	97.92%	98.95%
WS B	98.71%	93.7%	96.14%
WS C	100%	99.77%	99.88%
HM A	97.92%	96.83%	97.37%
HM B	96.43%	100%	98.18%
HM C	100%	99.77%	99.88%

**Table 4:** Ablation experiments verify the impact of each module on MPL-CNN.

CNN	LSTM	Dropout	Accuracy	P
YES	NO	NO	83.63%	86.46%
YES	NO	YES	90.62%	88.54%
NO	YES	NO	94.2%	87.5%
NO	YES	YES	97.67%	90.62%
YES	YES	NO	97.92%	95.83%
YES	YES	YES	99.22%	97.54%

## Data Availability

Data is contained within the article.
